# Red blood cell distribution width is associated with coronary artery disease in latent tuberculosis infection: a machine learning-based study

**DOI:** 10.3389/fcimb.2026.1836144

**Published:** 2026-08-14

**Authors:** Feng Sun, Yuqiang Zhou, Haiping Ma, Mei Ye, Yining Yang

**Affiliations:** 1 Department of Respiratory Medicine, The First Affiliated Hospital of Xinjiang Medical University, Urumqi, China; 2 Department of Cardiology, The Fifth Affiliated Hospital of Xinjiang Medical University, Urumqi, China; 3 First Clinical Medical College, Xinjiang Medical University, Urumqi, China; 4 Imaging Center, The First Affiliated Hospital of Xinjiang Medical University, Urumqi, China; 5 Department of Cardiology, People’s Hospital of Xinjiang Uyghur Autonomous Region, Urumqi, China; 6 Xinjiang Key Laboratory of Cardiovascular Homeostasis and Regeneration Research, Urumqi, China

**Keywords:** chronic inflammation (CI), coronary artery disease (CAD), latent tuberculosis infection (LTBI), machine learning, red blood cell distribution width (RDW)

## Abstract

**Background:**

Latent tuberculosis infection (LTBI) is associated with an increased risk of coronary artery disease (CAD), potentially mediated by systemic inflammation. Traditional lipid-based risk models may have limited predictive performance in tuberculosis-affected populations.

**Objectives:**

This study aimed to identify CAD risk factors in individuals with LTBI and to develop and externally validate a predictive model using a Boruta–LASSO machine learning approach.

**Methods:**

In this dual-center retrospective case–control study, patients who underwent both coronary CT angiography (CCTA) and interferon-γ release assay (IGRA) were enrolled. Cases were patients with CAD diagnosed by CCTA; controls were those without CAD. Candidate predictors were selected using Boruta and LASSO regression, then incorporated into a multivariable logistic regression model to construct a nomogram, which was externally validated.

**Results:**

Among 862 screened records, 616 participants were enrolled (derivation cohort, *n* = 453; validation cohort, *n* = 163). Age, red blood cell distribution width (RDW), and hemoglobin A1c (HbA1c) were identified as independent predictors and incorporated into the nomogram. The model achieved an area under the receiver operating characteristic curve (AUC) of 0.783 (MAE 0.285) in the derivation cohort and 0.738 (MAE 0.303) in the external validation cohort, with favorable net clinical benefit. A significant RDW–LTBI interaction was associated with a 1.737-fold (95% CI 1.092–2.877, *P* = 0.025) increase in CAD risk. After Benjamini–Hochberg correction (FDR < 0.05), RDW was positively correlated with interleukin-6 (IL-6; *r* = 0.345), C-reactive protein (CRP; *r* = 0.251), serum amyloid A (SAA; *r* = 0.238), and monocyte-to-lymphocyte ratio (MLR; *r* = 0.214).

**Conclusion:**

A nomogram for predicting CAD risk in LTBI-positive individuals was developed and externally validated using three routine clinical variables. A significant multiplicative interaction between RDW and LTBI was identified, and positive correlations between RDW and pro-inflammatory markers were observed specifically in LTBI-positive patients. These findings are consistent with the hypothesis that LTBI-associated chronic inflammation may contribute to CAD risk and that this low-cost tool may facilitate early risk stratification in resource-limited settings.

## 1 Introduction

Latent tuberculosis infection (LTBI) is defined as a persistent immune response to *Mycobacterium tuberculosis* without clinical symptoms or transmissibility. According to the World Health Organization, approximately one-quarter of the global population is estimated to have LTBI (World Health Organization, 2024). In China, a country with one of the highest tuberculosis prevalence rates worldwide, the LTBI burden is estimated to exceed 350 million individuals (Cui et al., 2020). Accumulating epidemiological evidence has established LTBI as an independent risk factor for coronary artery disease (CAD). A meta-analysis (Sumbal et al., 2023) demonstrated that the risk of CAD is substantially elevated in LTBI-positive compared with LTBI-negative individuals (odds ratio 2.08, 95% CI 1.45–2.98). Furthermore, compared with uninfected individuals, those with LTBI have a significantly higher risk of CAD (hazard ratio 1.52), an association that remains independent of traditional cardiovascular risk factors (Huaman et al., 2021; Alsayed Hasanain et al., 2018). An independent cohort study further reported that LTBI remains independently associated with acute myocardial infarction after adjusting for traditional cardiovascular risk factors (Huaman et al., 2018). Importantly, cardiovascular disease—rather than tuberculosis reactivation—has emerged as the leading cause of death among individuals across the tuberculosis infection spectrum, accounting for the majority of excess long-term mortality in this population (Sumbal et al., 2023). These data underscore an urgent public health need to identify LTBI individuals at elevated cardiovascular risk and to intervene before clinical events occur.

Despite this need, CAD risk prediction in the LTBI population faces unique challenges. Traditional risk assessment tools, such as the Framingham Risk Score, rely heavily on serum lipid profiles. However, patients with tuberculosis have been shown to exhibit significantly reduced levels of total cholesterol, low-density lipoprotein cholesterol, and high-density lipoprotein cholesterol compared with healthy controls (Kubalová et al., 2025), potentially attenuating the discriminative accuracy of lipid-based models in LTBI populations. Moreover, advanced cardiovascular risk assessment tools—such as coronary artery calcium scoring and carotid intima–media thickness measurement—are often unavailable in primary care and community hospital settings in LTBI-endemic regions. Therefore, a prediction model employing routinely available, low-cost clinical indicators would be better suited for broad implementation in resource-limited settings where the LTBI burden is highest.

From a mechanistic perspective, the LTBI–CAD link is thought to be mediated primarily through persistent immune activation and chronic low-grade inflammation (Djaharuddin et al., 2023). Systemic inflammatory markers—including C-reactive protein (CRP), interleukin-6 (IL-6), and leukocyte subset ratios—have been consistently implicated in the initiation and progression of atherosclerosis (Libby, 2021). Among these, red blood cell distribution width (RDW), a routinely reported hematological parameter reflecting erythrocyte volume heterogeneity, has emerged as a robust prognostic indicator across a spectrum of cardiovascular diseases (Danese et al., 2015; Shah et al., 2017; Li et al., 2023). Elevated RDW is thought to integrate multiple pro-atherosclerotic signals—including inflammation-induced suppression of erythropoiesis, oxidative stress, and iron dysregulation (Fraenkel, 2015; Li et al., 2023). However, whether RDW contributes to CAD risk prediction specifically in the LTBI population, and whether its predictive value is modified by LTBI status, has not been examined.

In this dual-center retrospective study, we enrolled patients who underwent both interferon-γ release assay (IGRA) and coronary computed tomography angiography (CCTA). Demographic characteristics, traditional cardiovascular risk factors, routine blood parameters, and inflammation-related indices were systematically collected. We aimed to identify risk factors for coronary atherosclerosis in the LTBI population, to develop and validate a CAD risk nomogram based on routinely available clinical indicators, and to explore the associations between systemic inflammatory markers and CAD, thereby providing new insights for mechanistic research and clinical screening of LTBI-associated cardiovascular risk.

## 2 Methods

### 2.1 Study design and population

This was a dual-center retrospective cohort study. We enrolled inpatients aged ≥18 years who had completed IGRA), pulmonary computed tomography (CT), and CCTA during a single hospitalization. The derivation cohort A comprised patients from the First Affiliated Hospital of Xinjiang Medical University (January–December 2024), and the external validation cohort B comprised patients from the Fifth Affiliated Hospital (January 2020–December 2024). Exclusion criteria were active tuberculosis, acute infection within 30 days, malignancy, immunosuppression, major organ failure, or acute cerebrovascular disease. To mitigate selection bias, only the first eligible admission was analyzed for patients with multiple hospitalizations.

### 2.2 Ethics approval and consent to participate

This study was approved by the Ethics Committee of Xinjiang Medical University (Application No. K202507-70). Written informed consent was waived because the study used only anonymized retrospective data. All procedures were conducted in accordance with the ethical principles of the *Declaration of Helsinki* and the *Ethical Review Measures for Life Science and Medical Research Involving Human Subjects* of China.

### 2.3 Diagnostic criteria

#### 2.3.1 Latent tuberculosis infection

LTBI was defined as a positive IGRA result in the absence of active tuberculosis, as confirmed by chest CT and negative sputum etiological tests for *M. tuberculosis*. Because the two participating centers used different IGRA formats, test-specific positivity thresholds were applied. In cohort A (QFT-Plus), positivity was defined as either IGRA1 or IGRA2 ≥0.35 IU/mL above the nil value (with nil < 8.0 IU/mL) and mitogen ≥0.5 IU/mL. In cohort B (QFT-GIT), positivity was defined as a single-tube interferon-γ level ≥0.35 IU/mL above the nil value, with the same validity criteria (nil < 8.0 IU/mL and mitogen ≥ 0.5 IU/mL).

#### 2.3.2 Coronary artery disease

CAD was diagnosed by the presence of coronary atherosclerotic plaques on CCTA. All CCTA findings were independently interpreted by a study radiologist who was blinded to clinical data. In cases where the study radiologist’s findings differed from the original CCTA reports in the medical records, a third expert panel adjudicated the final diagnosis.

### 2.4 Grouping strategy

Participants were assigned to two parallel group classifications: CAD versus non-CAD based on CCTA findings and LTBI versus non-LTBI based on the diagnostic criteria defined in Section 2.3.1.

### 2.5 Data collection

Data were systematically extracted from electronic medical records (EMR), including demographic characteristics (age, sex), discharge diagnoses, comorbidities, laboratory test results, chest CT imaging findings, and CCTA reports. The same data definitions and collection protocols were applied at both study centers to ensure consistency.

### 2.6 Sample size estimation

Sample size was estimated using the events per variable (EPV) criterion. At the study design stage, up to five candidate predictors were anticipated. With a conservative EPV threshold of 10 events per variable, at least 50 CAD events were required in the LTBI group. Published estimates of CAD prevalence in LTBI populations vary widely, ranging from 9% for obstructive disease (Huaman et al., 2021) to 84% for any coronary atherosclerosis (Khoufi, 2021). In the actual analysis, three independent predictors (age, HbA1c, and RDW) were retained. The observed CAD prevalence in the LTBI group of cohort A was 76.8%, yielding 182 CAD events among 237 LTBI participants. This corresponded to 61 events per variable—six times the recommended minimum—confirming adequate statistical power for model fitting. The external validation cohort B included 71 LTBI participants with 52 CAD events (17 events per variable), providing sufficient power for model evaluation.

### 2.7 Statistical analysis and prediction model development

#### 2.7.1 Descriptive statistics and group comparisons

All analyses were performed using SPSS software, v. 25 (IBM) and R (version 4.4.3; R Foundation for Statistical Computing, Vienna, Austria). A two-sided *P <*0.05 was considered statistically significant. Normality was assessed using the Shapiro–Wilk test. Variables with a normal distribution are presented as mean ± SD; non-normally distributed variables are presented as median with IQR. The independent-samples *t*-test was used for normally distributed variables, the Mann–Whitney *U* test for non-normally distributed variables, and the chi-square test or Fisher’s exact test for categorical variables. Categorical variables are expressed as frequencies. ORs with 95% CIs were derived from logistic regression coefficients. All figures were generated using the *ggplot2* package in R.

#### 2.7.2 Handling of missing data

For continuous variables, multiple imputation by chained equations (MICE) was performed using the *mice* package in R, with 5 imputed datasets and 20 iterations per dataset. For categorical variables, mode imputation was used. Variables with a missing rate exceeding 20% were excluded from the analysis. All imputation and feature selection procedures were performed exclusively within the training set (cohort A), with the validation set (cohort B) held out and not used in any model development step to prevent data leakage.

#### 2.7.3 Feature selection and model development

In LTBI participants from cohort A, candidate variables were independently screened using two parallel feature selection methods: the Boruta algorithm and least absolute shrinkage and selection operator (LASSO) penalization regression with 10-fold cross-validation (*λ* selected at 1 standard error from the minimum [*λ*_1se_]). The final set of predictors was defined as the intersection of variables selected by both methods. These variables were then entered into a multivariable logistic regression model; only those with *P <*0.05 (Wald test) in the multivariable model were retained and used to build the final nomogram. Given the moderate class imbalance (CAD-to-non-CAD ratio approximately 3.3:1 in the LTBI-positive training set) and the adequate events-per-variable ratio (60.7 with three predictors), no additional class rebalancing was applied, as logistic regression estimates are generally robust under these conditions.

#### 2.7.4 Model performance and validation

The nomogram was internally validated using 1,000 bootstrap resamples. The apparent C-index (the C-index estimated directly from the training set) was calculated, and the mean optimism (the average difference between performance in bootstrap samples and performance in the original sample) was subtracted to obtain the optimism-corrected C-index. Calibration was assessed using calibration curves and the Hosmer–Lemeshow test. Decision curve analysis (DCA) was performed to evaluate the clinical net benefit. The final model was then applied to LTBI individuals in cohort B for external validation, with performance assessed by the C-index, calibration curves, the Hosmer–Lemeshow test, and DCA.

#### 2.7.5 Effect modification analysis

To evaluate whether the association between each predictor and CAD differed by LTBI status, multiplicative interaction terms (LTBI × predictor) were added to the logistic regression model in the full cohort A. A Wald *χ*² test *P <*0.05 was considered evidence of effect modification. Significant interactions were visualized as predictive probability curves, with 95% bootstrap percentile confidence intervals.

#### 2.7.6 Exploratory correlation analysis

For mechanistic exploration, Spearman rank correlation coefficients were computed between the LTBI-associated predictor (RDW) and systemic inflammatory markers. To control for multiple comparisons, *P*-values were adjusted using the Benjamini–Hochberg false discovery rate (FDR) procedure, with an FDR threshold of <0.05 considered statistically significant. Only significant associations are reported.

## 3 Results

### 3.1 Subject flow and baseline characteristics

**Figure 1:**
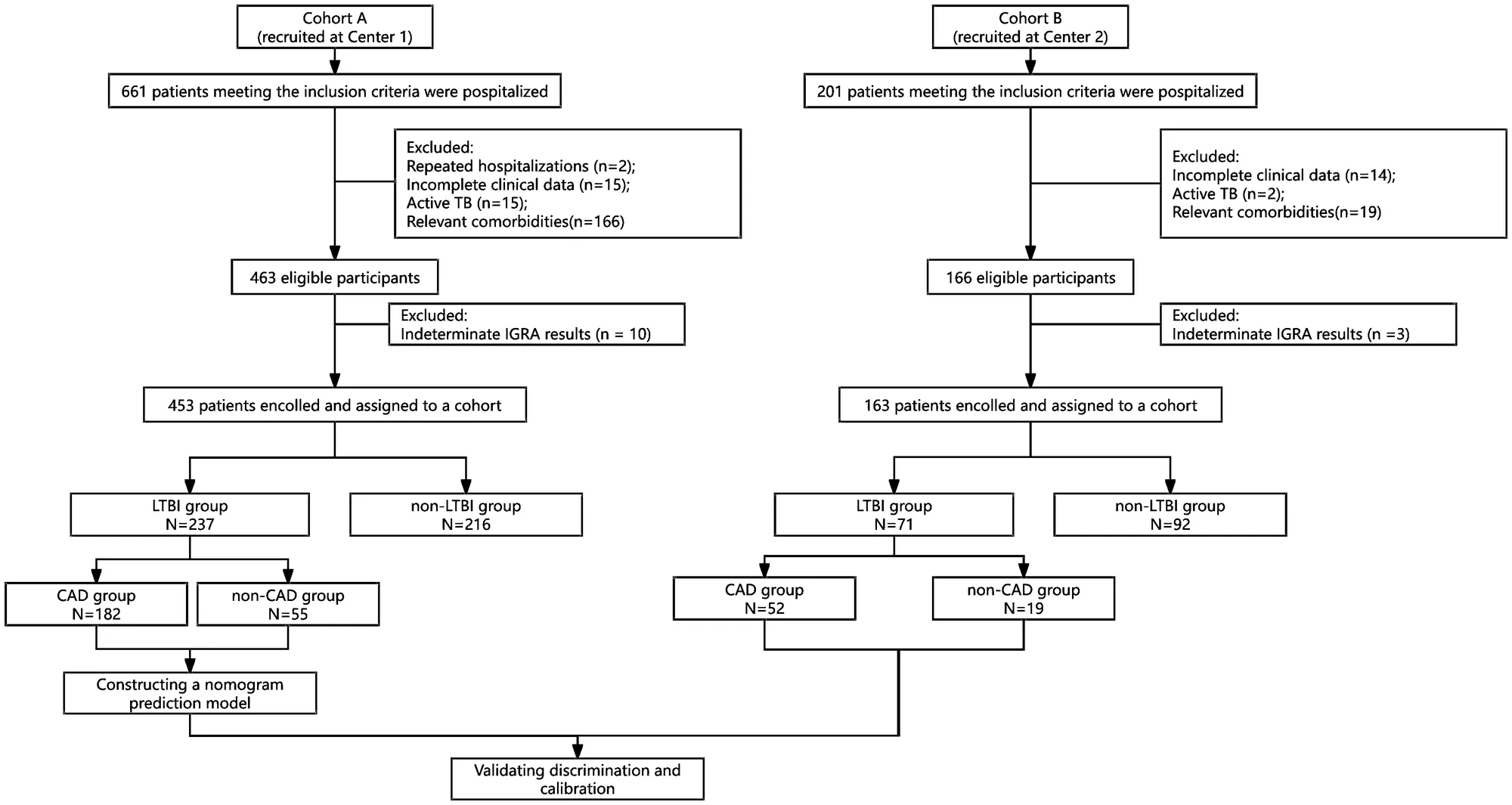
Participant flowchart: cohort A (derivation) and cohort B (external validation). Cohort A (derivation) was recruited from the First Affiliated Hospital of Xinjiang Medical University (January–December 2024). Of 661 initially screened records, 208 were excluded, leaving 453 patients included in the analysis (LTBI-positive, *n* = 237; LTBI-negative, *n* = 216). Cohort B (external validation) was recruited from the Fifth Affiliated Hospital of Xinjiang Medical University (January 2020–December 2024). Of 201 initially screened records, 38 were excluded, leaving 163 patients included in the analysis (LTBI-positive, *n* = 71; LTBI-negative, *n* = 92). Exclusion criteria: active tuberculosis, acute infection within 30 days prior to enrollment, malignancy, immunosuppression, major organ failure, acute cerebrovascular disease, and multiple hospitalizations (only the first eligible admission was analyzed).

A total of 661 patients from cohort A and 201 from cohort B were initially screened, of whom 453 (cohort A) and 168 (cohort B) were included after applying the inclusion and exclusion criteria (**Figure 1**). Age, total cholesterol (TC), and low-density lipoprotein cholesterol (LDL-C) were normally distributed; all other continuous variables were non-normally distributed. In cohort A, the mean age was 63.55 ± 11.86 years, with 256 men (56.5%) and 197 women (43.5%).

Baseline characteristics of LTBI (*n* = 237) and non-LTBI (*n* = 216) participants in cohort A are summarized in **Table 1**. The two groups were comparable in age, sex, smoking status, education level, occupation, and the majority of comorbidities (all *P* > 0.05). Among clinical laboratory parameters, no significant differences were observed in lipid profiles [TC, triglycerides (TG), high-density lipoprotein cholesterol (HDL-C), LDL-C] or inflammatory cytokines [IL-6, serum amyloid A (SAA), CRP] (all *P* > 0.05). However, the LTBI group had significantly higher BMI, eosinophil count, lymphocyte count, and HbA1c (all *P* < 0.05). Detailed comparisons are shown in **Table 1** and **Supplementary Table 1** (in the **Supplementary Materials**).

**Table 1:** Baseline characteristics of cohort A by LTBI and CAD status.

Indexes	LTBI (*n* = 237)	Control (*n* = 216)	*P*-value	CAD (*n* = 345)	Non-CAD (*n* = 108)	*P*-value
Age (years)	63.41 ± 10.75	63.71 ± 12.99	0.786	66.14 ± 10.81	55.31 ± 11.32	<0.001
Sex, *n* (%)						
Male	125 (52.7%)	131 (60.6%)	0.090	213 (61.7%)	43 (39.8%)	<0.001
Female	112 (47.3%)	85 (39.4%)		132 (38.3%)	65 (60.2%)	
BMI (kg/m²)	26.00 (23.00, 28.00)	24.00 (22.00, 27.00)	0.006	25.00 (22.00, 28.00)	25.00 (23.00, 28.00)	0.501
Smoking, *n* (%)			0.289			0.358
Never	179 (75.5)	149 (69.0)		244 (70.7)	84 (77.8)	
Current	22 (9.3)	24 (11.1)		37 (10.7)	9 (8.3)	
Former	36 (15.2)	43 (19.9)		64 (18.6)	15 (13.9)	
Education, *n* (%)			0.347			0.104
Primary or below	82 (34.6)	61 (28.2)		111 (32.2)	32 (29.6)	
Middle/high school	93 (39.2)	93 (43.1)		148 (42.9)	38 (35.2)	
College or above	62 (26.2)	62 (28.7)		86 (24.9)	38 (35.2)	
Occupation, *n* (%)			0.836			0.020
Unemployed/retire	123 (51.9)	112 (51.9)		184 (53.3)	51 (47.2)	
Self-employed/flexible	81 (34.2)	70 (32.4)		119 (34.5)	32 (29.6)	
Formally employed	33 (13.9)	34 (15.7)		42 (12.2)	25 (23.1)	
Comorbidities, *n* (%)						
CAD	182 (76.8%)	163 (75.5%)	0.740			
Non-CAD	55 (23.2%)	53 (24.5%)				
HP	121 (51.1%)	111 (51.4%)	0.943	198 (57.4%)	34 (31.5%)	<0.001
DM	60 (25.3%)	47 (21.7%)	0.373	96 (27.8%)	11 (10.2%)	<0.001
CRD	72 (30.4%)	70 (32.4%)	0.642	115 (33.3%)	27 (25.0%)	0.103
AF	15 (6.3%)	13 (6.0%)	0.891	25 (7.2%)	3 (2.8%)	0.092
HF	26 (11.0%)	23 (10.6%)	0.912	42 (12.2%)	7 (6.5%)	0.096
RF	13 (5.5%)	20 (9.3%)	0.123	26 (7.5%)	7 (6.5%)	0.713
WBC (×10^9^/L)	6.66 (5.56, 8.35)	6.45 (5.25, 8.00)	0.124	6.62 (5.45, 8.31)	6.23 (5.38, 8.19)	0.526
N (×10^9^/L)	3.96 (3.16, 5.48)	3.98 (2.93, 5.35)	0.522	4.14 (3.10, 5.42)	3.55 (2.91, 5.15)	0.116
L (×10^9^/L)	1.83 (1.38, 2.31)	1.63 (1.25, 2.10)	0.008	1.64 (1.25, 2.17)	1.90 (1.52, 2.33)	0.002
M (×10^9^/L)	0.54 (0.42, 0.70)	0.52 (0.40, 0.68)	0.202	0.54 (0.42, 0.69)	0.49 (0.40, 0.65)	0.151
E (×10^9^/L)	0.12 (0.07, 0.21)	0.11 (0.06, 0.18)	0.042	0.12 (0.07, 0.21)	0.11 (0.07, 0.18)	0.884
Hb (g/L)	133.00 (118.00, 145.00)	134.00 (123.00, 147.00)	0.214	133.00 (121.00, 146.00)	134.00 (121.50, 144.25)	0.966
RDW (%)	13.00 (12.50, 13.70)	13.10 (12.50, 13.80)	0.642	13.20 (12.60, 13.90)	12.75 (12.28, 13.40)	<0.001
PLT (×10^12^/L)	235.00 (193.00, 284.00)	217.00 (177.75, 279.00)	0.058	222.00 (183.00, 274.00)	256.50 (204.25, 316.00)	0.001
PDW	10.80 (9.60, 12.20)	10.65 (9.70, 11.90)	0.713	10.70 (9.60, 12.10)	10.80 (9.70, 12.03)	0.699
TC (mmol/L)	4.02 ± 1.03	3.92 ± 1.00	0.309	3.89 ± 1.02	4.24 ± 0.94	0.002
TG (mmol/L)	3.70 (2.59, 4.50)	3.42 (2.33, 4.39)	0.070	3.42 (2.37, 4.42)	4.04 (2.97, 4.70)	0.006
LDL-C (mmol/L)	2.67 ± 0.85	2.56 ± 0.84	0.149	2.55 ± 0.87	2.82 ± 0.74	0.004
HDL-C (mmol/L)	0.95 (0.80, 1.13)	0.98 (0.81, 1.15)	0.333	0.94 (0.79, 1.11)	1.08 (0.88, 1.23)	<0.001
CRP (mg/L)	7.20 (5.00, 15.30)	6.90 (5.00, 14.93)	0.765	8.10 (5.00, 17.30)	5.75 (5.00, 9.23)	0.002
IL-6 (pg/mL)	5.09 (2.67, 12.02)	5.45 (2.54, 14.88)	0.745	5.78 (2.85, 16.30)	3.75 (2.06, 6.85)	0.002
SAA	5.50 (2.86, 18.48)	6.57 (3.38, 25.65)	0.175	6.66 (3.25, 28.88)	5.02 (2.56, 9.40)	0.003
HbA1c	6.00 (5.54, 6.79)	5.80 (5.41, 6.49)	0.047	5.98 (5.58, 6.80)	5.64 (5.25, 6.03)	<0.001
>6%	112 (47.257)	80 (37.037)	0.028	164 (47.536)	28 (25.926)	<0.001
≤6%	125 (52.743)	136 (62.963)		181 (52.464)	80 (74.074)	
IGRA (IU/mL)	3.22 (1.18, 8.59)	0.02 (0.00, 0.07)	<0.001	0.50 (0.03, 3.40)	0.25 (0.02, 3.21)	0.286
IGRA-N (IU/mL)	0.11 (0.05, 0.37)	0.05 (0.03, 0.09)	<0.001	0.07 (0.04, 0.19)	0.07 (0.04, 0.16)	0.768
IGRA-P (IU/mL)	39.25 (29.88, 56.93)	35.26 (28.28, 48.33)	0.021	36.22 (28.83, 51.92)	41.94 (30.59, 60.08)	0.025
IGRA%	0.09 (0.03, 0.19)	0.00 (0.00, 0.00)	<0.001	0.01 (0.00, 0.10)	0.01 (0.00, 0.08)	0.138

Age, TC, and LDL-C are normally distributed and presented as mean ± SD; all other continuous variables are presented as median (Q1, Q3). Categorical variables are expressed as *n* (%). CAD, coronary artery disease; HP, hypertension; DM, diabetes mellitus; CRD, chronic respiratory disease; AF, atrial fibrillation; HF, heart failure; RF, respiratory failure; WBC, white blood cell count; N, neutrophil count; L, lymphocyte count; M, monocyte count; E, eosinophil count; Hb, hemoglobin; RDW, red cell distribution width; PLT, platelets; PDW, platelet distribution width; TC, total cholesterol; TG, triglyceride; LDL-C, low-density lipoprotein cholesterol; HDL-C, high-density lipoprotein cholesterol; CRP, C-reactive protein; IL-6, interleukin-6; SAA, serum amyloid A; HbA1c, glycated hemoglobin; IGRA, γ-interferon release assay; IGRA-N, the results of the negative control group in the IGRA assay; IGRA-P, the results of the positive control group in the IGRA assay; IGRA%, IGRA-to-IGRA-P ratio.

### 3.2 Machine learning-based development and validation of a prediction model for LTBI-associated CAD

**Figure 2:**
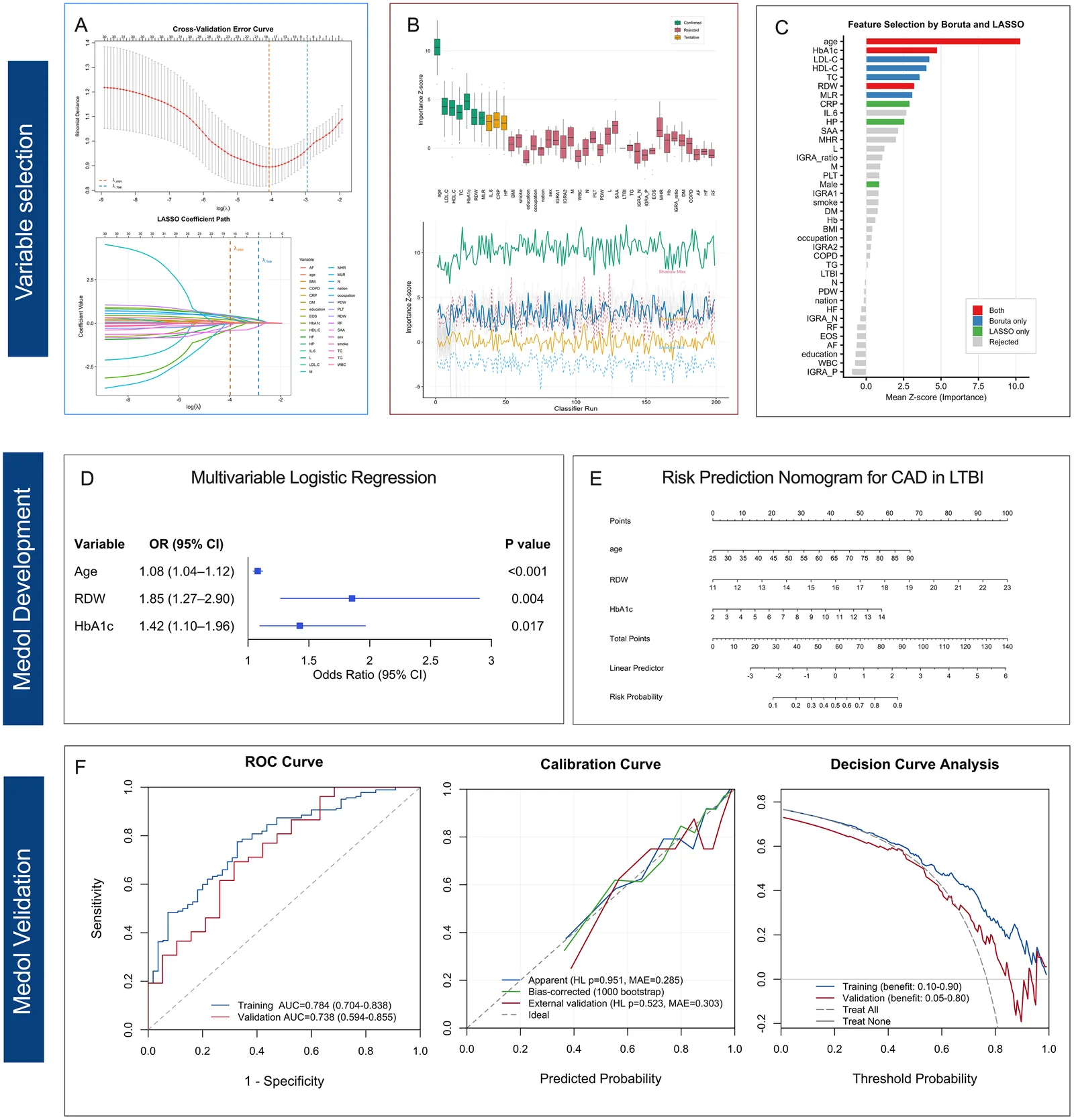
Variable screening, model development, and validation workflow. **(A)** LASSO regression with 10-fold cross-validation: cross-validation error curve (top) and coefficient path plot (bottom). The red dashed line indicates *λ*_min_ = 0.0187; the blue dashed line indicates *λ*_1se_ = 0.063. **(B)** Boruta feature selection: *Z*-score distributions of confirmed, tentative, and rejected attributes (top) and importance trend curves across Boruta iterations (bottom). **(C)** Feature selection by Boruta and LASSO. **(D)** Multivariable logistic regression results: forest plot of odds ratios with 95% confidence intervals for age, HbA1c, and RDW in LTBI-positive participants. **(E)** Nomogram for estimating CAD probability in LTBI-positive individuals, integrating age (years), HbA1c (%), and RDW (%). **(F)** Model validation: receiver operating characteristic (ROC) curves (left), calibration plots (middle), and decision curve analysis (DCA, right). Red lines, derivation cohort; blue lines, external validation cohort. AUC, area under the ROC curve; MAE, mean absolute error.

After LTBI stratification, intergroup differences are listed in **Table 2**. In the LTBI group, CAD patients were older and had higher RDW, HbA1c, and CRP and lower HDL-C (all *P* < 0.05; **Table 2**). In the non-LTBI group, CAD patients exhibited significantly lower BMI (*P* = 0.004), likely reflecting reverse causation or the known obesity paradox in cardiovascular disease, and had different occupational distribution (*P* = 0.001), with formally employed individuals being less likely to have CAD. In the LTBI-positive training set, a Boruta–LASSO pipeline was applied: Boruta (10,000 permutations) retained age, MLR, LDL-C, HDL-C, TC, RDW, and HbA1c, while LASSO (10-fold cross-validation, *λ*_1se_ = 0.063) retained age, male sex, CRP, RDW, HP, and HbA1c. The intersection of both methods—age, RDW, and HbA1c—was entered into a multivariable logistic regression model (*χ*² = 48.57, *P* < 0.0001). Multivariable logistic regression identified age (per 1-year increase: OR = 1.08, 95% CI 1.04–1.12, *P* < 0.001), HbA1c (per 1% increase: OR = 1.42, 95% CI 1.10–1.96, *P* = 0.017), and RDW (per 1% increase: OR = 1.85, 95% CI 1.27–2.90, *P* = 0.004) as independent predictors of CAD in the LTBI group (**Supplementary Table 3**). These three variables were used to construct the nomogram (**Figure 2C**).

**Table 2:** Differences in clinical parameters between CAD and non-CAD subgroups within the LTBI and non-LTBI cohorts.

Indexes	LTBI (*n* = 237)			Non-LTBI (*n* = 216)		
	CAD (*n* = 182)	Non-CAD (*n* = 55)	*P*	CAD (*n* = 163)	Non-CAD (*n* = 54)	*P*
Age (years)	65.43 ± 9.97	56.71 ± 10.59	<0.001	66.92 ± 11.65	53.85 ± 11.96	<0.001
Male, *n* (%)	104 (57.1%)	21 (38.9%)	0.014	109 (66.9%)	22 (40.7%)	0.001
BMI (kg/m²)	26.00 (24.00, 29.00)	25.00 (22.00, 27.00)	0.066	24.00 (21.00, 27.00)	25.00 (24.00, 29.00)	0.004
Smoking, *n* (%)			0.344			0.590
Never	134 (73.6)	45 (81.8)		110 (67.5)	39 (73.6)	
Current	17 (9.3)	5 (9.1)		20 (12.3)	4 (7.5)	
Former	31 (17.0)	5 (9.1)		33 (20.2)	10 (18.9)	
Education, *n* (%)			0.704			0.023
Primary or below	61 (33.5)	21 (38.2)		50 (30.7)	11 (20.8)	
Middle/high school	74 (40.7)	19 (34.5)		74 (45.4)	19 (35.8)	
College or above	47 (25.8)	15 (27.3)		39 (23.9)	23 (43.4)	
Occupation, *n* (%)			0.982			0.001
Unemployed/retired	95 (52.2)	28 (50.9)		89 (54.6)	23 (43.4)	
Self-employed/flexible	62 (34.1)	19 (34.5)		57 (35.0)	13 (24.5)	
Formally employed	25 (13.7)	8 (14.5)		17 (10.4)	17 (32.1)	
Comorbidities, *n* (%)						
HP, *n* (%)	105 (57.7%)	16 (29.6%)	<0.001	93 (57.1%)	18 (33.3%)	0.003
DM, *n* (%)	53 (29.1%)	7 (13.0%)	0.014	43 (26.4%)	4 (7.4%)	0.007
CRD, *n* (%)	58 (31.9%)	14 (26.0%)	0.365	57 (35.0%)	13 (24.1%)	0.158
AF, *n* (%)	13 (7.1%)	2 (3.7%)	0.535	12 (7.4%)	1 (1.9%)	0.261
HF, *n* (%)	21 (11.5%)	5 (9.3%)	0.611	21 (12.9%)	2 (3.7%)	0.107
RF, *n* (%)	10 (5.5%)	3 (5.6%)	1.000	16 (9.8%)	4 (7.4%)	0.824
WBC (×10^9^/L)	6.80 (5.59, 8.38)	6.27 (5.37, 8.20)	6.80 (5.59, 8.38)	6.47 (5.25, 7.89)	6.14 (5.40, 8.06)	0.900
N (×10^9^/L)	4.03 (3.31, 5.52)	3.54 (2.88, 5.30)	4.03 (3.31, 5.52)	4.25 (2.94, 5.38)	3.65 (2.93, 5.01)	0.503
L (×10^9^/L)	1.73 (1.33, 2.26)	1.98 (1.56, 2.40)	1.73 (1.33, 2.26)	1.58 (1.18, 2.04)	1.86 (1.48, 2.19)	0.010
M (×10^9^/L)	0.55 (0.44, 0.70)	0.48 (0.38, 0.68)	0.55 (0.44, 0.70)	0.52 (0.40, 0.69)	0.49 (0.41, 0.64)	0.703
E (×10^9^/L)	0.12 (0.07, 0.22)	0.12 (0.08, 0.19)	0.12 (0.07, 0.22)	0.11 (0.05, 0.20)	0.10 (0.07, 0.16)	0.938
Hb (g/L)	133.00 (117.00, 144.75)	135.00 (121.00, 145.00)	0.514	134.00 (123.00, 147.50)	134.00 (122.00, 143.00)	0.558
RDW (%)	13.10 (12.60, 13.90)	12.60 (12.25, 13.30)	<0.001	13.20 (12.60, 13.85)	12.80 (12.30, 13.50)	0.068
PLT (×10^12^/L)	228.00 (189.50, 281.50)	258.00 (210.00, 312.50)	0.018	214.00 (170.50, 270.50)	249.00 (188.00, 322.00)	0.012
PDW	10.80 (9.60, 12.20)	10.80 (9.50, 12.25)	0.873	10.60 (9.70, 11.90)	10.90 (9.90, 11.80)	0.425
TC (mmol/L)	3.96 ± 1.06	4.24 ± 0.90	0.076	3.8 ± 1.0	4.3 ± 1.0	0.006
TG (mmol/L)	3.62 (2.57, 4.50)	3.99 (2.81, 4.63)	0.443	3.82 ± 0.98	4.25 ± 0.98	0.006
LDL-C (mmol/L)	2.61 ± 0.88	2.86 ± 0.70	0.054	3.18 (1.81, 4.16)	4.07 (3.09, 4.92)	0.002
HDL-C (mmol/L)	0.92 (0.79, 1.10)	1.06 (0.90, 1.20)	0.004	2.48 ± 0.86	2.77 ± 0.77	0.030
CRP (mg/L)	8.45 (5.00, 17.90)	5.30 (5.00, 9.40)	0.001	7.50 (5.00, 16.65)	6.50 (5.00, 8.70)	0.130
IL-6 (pg/mL)	5.71 (3.11, 15.95)	3.81 (1.75, 6.55)	0.002	6.10 (2.62, 17.65)	3.70 (2.22, 8.84)	0.055
SAA	6.09 (3.19, 25.50)	4.23 (2.38, 9.42)	0.011	7.47 (3.49, 31.39)	5.24 (2.93, 9.36)	0.106
HbA1c (%)	6.11 (5.60, 6.88)	5.70 (5.21, 6.05)	0.001	5.90 (5.55, 6.64)	5.62 (5.27, 6.01)	0.002
>6%	98	14	<0.001	66	14	0.065
≤6%	84	41		97	39	
IGRA (IU/mL)	3.24 (1.21, 9.03)	3.17 (1.17, 7.13)	0.632	0.02 (0.01, 0.09)	0.01 (0.00, 0.04)	0.082
IGRA-N (IU/mL)	0.11 (0.05, 0.33)	0.10 (0.06, 0.45)	0.836	0.05 (0.03, 0.09)	0.05 (0.03, 0.08)	0.671
IGRA-P (IU/mL)	38.42 (29.73, 55.74)	43.09 (31.07, 60.16)	0.195	34.37 (27.86, 46.10)	40.81 (30.66, 58.42)	0.064
IGRA%	0.09 (0.03, 0.21)	0.08 (0.03, 0.16)	0.256	0.00 (0.00, 0.00)	0.00 (0.00, 0.00)	0.032

Age, TC, and LDL-C are normally distributed and presented as mean ± SD; all other continuous variables are presented as median (Q1, Q3). Categorical variables are expressed as *n* (%). CAD, coronary artery disease; HP, hypertension; DM, diabetes mellitus; CRD, chronic respiratory disease; AF, atrial fibrillation; HF, heart failure; RF, respiratory failure; WBC, white blood cell count; N, neutrophil count; L, lymphocyte count; M, monocyte count; E, eosinophil count; Hb, hemoglobin; RDW, red cell distribution width; PLT, platelets; PDW, platelet distribution width; TC, total cholesterol; TG, triglyceride; LDL-C, low-density lipoprotein cholesterol; HDL-C, high-density lipoprotein cholesterol; CRP, C-reactive protein; IL-6, interleukin-6; SAA, serum amyloid A; HbA1c, glycated hemoglobin; IGRA, γ-interferon release assay; IGRA-N, the results of the negative control group in the IGRA assay; IGRA-P, the results of the positive control group in the IGRA assay; IGRA%, IGRA-to-IGRA-P ratio.

The nomogram demonstrated good discrimination, with an optimism-corrected C-index of 0.774 after 1,000 bootstrap resamples (apparent C-index = 0.783, optimism = 0.009). Internal calibration was excellent [Hosmer–Lemeshow *χ*² = 2.654, *df* = 8, *P* = 0.954; mean absolute error (MAE) = 0.285]. Decision curve analysis demonstrated net clinical benefit over “treat-all” or “treat-none” strategies across threshold probabilities of 0.10–0.90.

In the external validation cohort (cohort B, *n* = 71), the model maintained good discrimination [area under the receiver operating characteristic curve (AUC) = 0.738, 95% CI 0.602–0.874], with a modest decline of 0.045 from the derivation cohort. External calibration was satisfactory (Hosmer–Lemeshow *χ*² = 7.132, *df* = 8, *P* = 0.523; MAE = 0.303). Decision curve analysis confirmed net clinical benefit over default strategies across threshold probabilities of 0.26–0.99 (**Figure 2**).

### 3.3 Identification of RDW as a predictor modulated by LTBI status through interaction analysis

**Figure 3:**
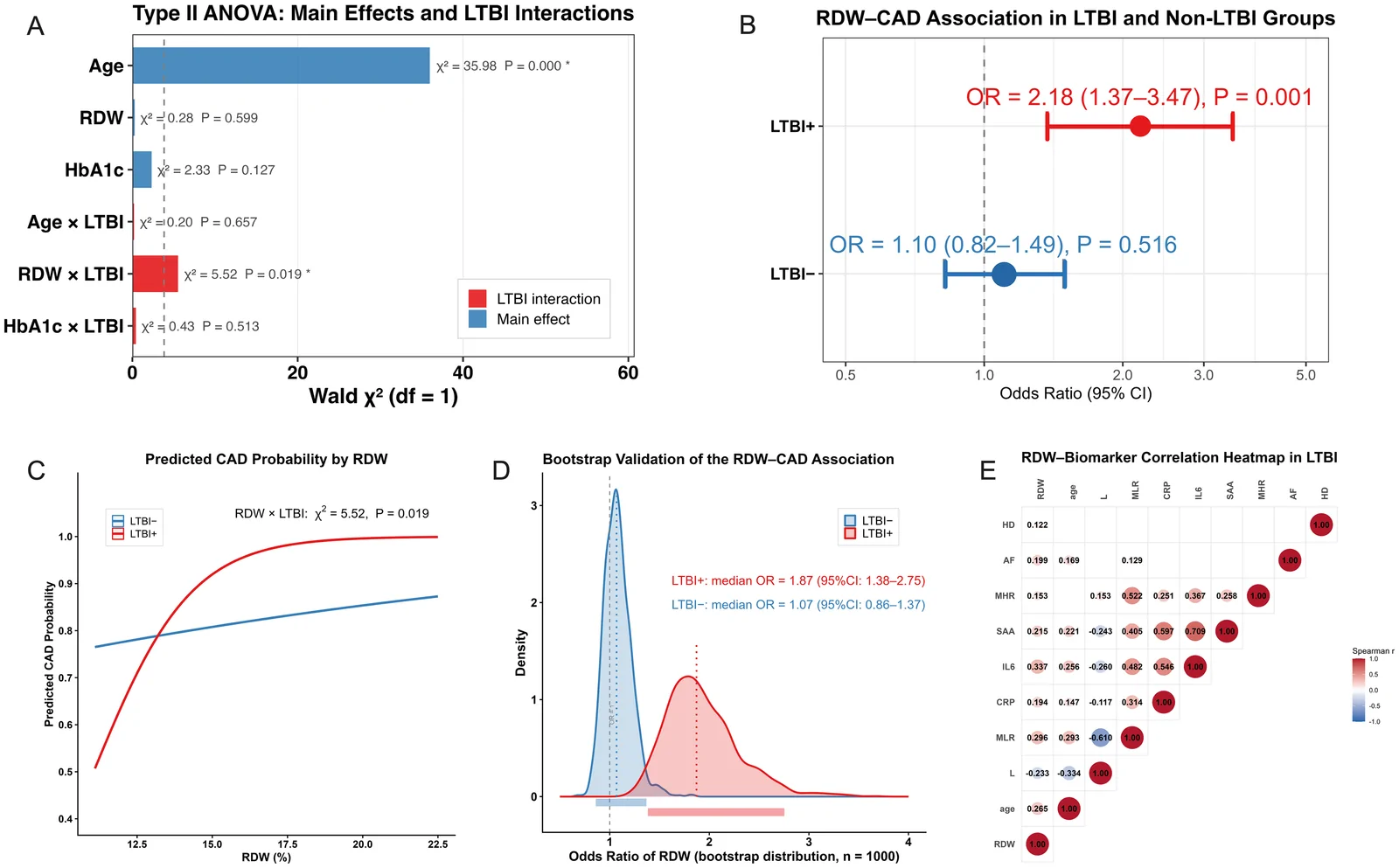
RDW–CAD associations and effect modification by LTBI status. **(A)** Type II analysis of deviance (Wald *χ*²) for multiplicative interaction terms (age × LTBI, RDW × LTBI, HbA1c × LTBI) simultaneously entered into the multivariable logistic model. Only the RDW × LTBI interaction was significant (*P* = 0.019). **(B)** Forest plot of RDW odds ratios stratified by LTBI status: LTBI-positive (OR = 2.18, 95% CI 1.37–3.49, *P* = 0.001) and LTBI-negative (OR = 1.10, 95% CI 0.82–1.48, *P* = 0.518). **(C)** Predicted CAD probability as a function of RDW, stratified by LTBI status, with age and HbA1c held at their medians. Shaded bands represent 95% bootstrap percentile confidence intervals. **(D)** Bootstrap density distributions (1,000 resamples) of the RDW odds ratio within each LTBI stratum. The LTBI-positive distribution (right-shifted) is centered above the null, while the non-LTBI distribution overlaps the null line (OR = 1.0). **(E)** Spearman correlation heat map between RDW and systemic inflammatory markers in LTBI-positive participants. Only variables with FDR <0.05 after Benjamini–Hochberg correction are shown. Color intensity reflects correlation strength; red denotes positive correlations, and blue denotes negative correlations. CRP, C-reactive protein; IL-6, interleukin-6; MLR, monocyte-to-lymphocyte ratio; SAA, serum amyloid A.

To test whether age, RDW, and HbA1c differentially influence CAD risk according to LTBI status, we introduced multiplicative interaction terms (age × LTBI, RDW × LTBI, HbA1c × LTBI) simultaneously into the multivariable logistic model. Type II analysis of deviance revealed that only the RDW × LTBI interaction was statistically significant (Wald *χ*² = 5.52, *df* = 1, *P* = 0.019; **Figure 3A**), whereas the age × LTBI and HbA1c × LTBI interactions showed no evidence of effect modification (both *P* > 0.05). Among the main effects, age was the dominant predictor (Wald *χ*² = 35.98, *P* < 0.001), while RDW and HbA1c showed no significant global effects.

To further characterize this interaction, we conducted LTBI-stratified logistic regression and visualized the results using a forest plot (**Figure 3B**). In the non-LTBI group, RDW showed no significant association with CAD (OR = 1.10, 95% CI 0.82–1.48, *P* = 0.518). In contrast, among the LTBI group, each 1% increment in RDW was independently associated with a 2.18-fold increase in the odds of CAD (OR = 2.18, 95% CI 1.37–3.49, *P* = 0.001). The interaction plot (**Figure 3C**) further illustrated the diverging risk trajectories: when age and HbA1c were held at their medians, the predicted probability of CAD rose steeply with increasing RDW in the LTBI group, whereas the non-LTBI group displayed a flat slope across the entire RDW range.

To assess the robustness of the stratified estimates, we performed 1,000 bootstrap resamples of the RDW effect within each LTBI stratum (**Figure 3D**). The bootstrap density distribution for the LTBI group was centered at an OR substantially above 1.0, with a narrow and right-shifted 95% confidence interval, indicating a stable and reproducible positive association. In contrast, the non-LTBI distribution was centered near the null (OR = 1.0) and broadly overlapped the null line, confirming the absence of an RDW effect in this group. The non-overlapping bootstrap distributions further reinforced the significant effect modification by LTBI status. Collectively, these analyses established RDW as a predictor modulated by LTBI status through interaction analysis.

### 3.4 Correlation analysis between RDW and other variables

In the LTBI group, Spearman correlation analysis with Benjamini–Hochberg correction (FDR < 0.05) identified significant positive associations between RDW and multiple inflammatory biomarkers, including IL-6 (*r* = 0.345), CRP (*r* = 0.251), SAA (*r* = 0.238), MLR (*r* = 0.214), and age (*r* = 0.223). These correlations, although modest in magnitude, consistently link RDW to systemic inflammation in LTBI individuals. The significant correlations are displayed in the heat map (**Figure 3E**), and the complete correlation matrix is provided in **Supplementary Table 4**. These correlations are consistent with the hypothesis that RDW-associated CAD risk in LTBI may, in part, reflect the cumulative hematological consequences of chronic low-grade inflammation, rather than the activity of a single dominant cytokine pathway.

## 4 Discussion

Despite the elevated cardiovascular risk documented in LTBI, a dedicated risk stratification tool for this population is still lacking. In the present study, we developed and externally validated a CAD risk prediction nomogram that, to our knowledge, is the first specifically designed for LTBI-positive individuals. Prior studies have identified LTBI as an independent risk factor for CAD, including acute myocardial infarction, typically by including IGRA status as a binary covariate in conventional regression models. Our nomogram, integrating age, RDW, and HbA1c, achieved an AUC of 0.783 in the derivation cohort and 0.738 in the external validation cohort, with favorable calibration and net clinical benefit. Notably, we identified a significant multiplicative interaction between RDW and LTBI, a finding not previously reported. These results indicate that RDW carries incremental predictive value for CAD risk that is specifically amplified in the context of LTBI.

### 4.1 Methodological innovations and clinical applicability

This study has several methodological strengths. A two-center design with different IGRA platforms (QFT-Plus for derivation; QFT-GIT for external validation) minimized center-specific bias and demonstrated cross-platform generalizability. A complementary two-stage variable selection strategy was adopted, in which Boruta identified robust features and LASSO regression applied penalized regularization. The intersection of these two methods yielded a parsimonious three-variable model, thereby reducing the risk of overfitting. Rather than employing a machine learning algorithm, we used conventional logistic regression to build the final model and presented it as a nomogram, ensuring full transparency in how each predictor contributes to risk estimation. Model performance was assessed across discrimination, calibration, and clinical utility, and the nomogram was externally validated in an independent cohort. Clinically, all three predictors are derived from routine assessments at no incremental cost, making the nomogram implementable in primary care settings where LTBI burden is the highest and advanced cardiovascular imaging is unavailable. Decision curve analysis confirmed net benefit across a broad range of threshold probabilities.

### 4.2 RDW as a predictor modulated by LTBI status and mechanistic insights

The most notable finding of this study is the significant multiplicative interaction between RDW and LTBI (OR 1.737, 95% CI 1.092–2.877, *P* = 0.025). This finding indicates that the same increment in RDW confers a substantially greater increase in CAD risk among LTBI-positive individuals than among their LTBI-negative counterparts. LTBI thus acts as an effect modifier rather than a confounder or mediator: it does not eliminate the RDW–CAD association but amplifies it. RDW has been recognized as an independent prognostic marker across diverse cardiovascular conditions, including heart failure (Noordermeer et al., 2026), coronary lesion severity (Zavragiu et al., 2025), and chronic kidney disease (Kimura et al., 2023). A systematic review and meta-analysis further confirmed that elevated RDW is independently associated with increased all-cause and cardiovascular mortality in patients with established CAD (Su et al., 2014). However, the prognostic value of RDW in CAD remains debated. Some studies have reported that the association between RDW and cardiovascular outcomes is substantially attenuated after adjusting for hemoglobin, iron status, and renal function, suggesting that RDW may partly reflect confounding by comorbidities rather than representing an independent causal pathway (Tonelli et al., 2008; Perlstein et al., 2009). Moreover, whether chronic infection status modifies the RDW–CAD relationship has not been previously examined. Our results provide the first evidence that LTBI-specific pathophysiology magnifies the cardiovascular risk signal captured by RDW, and the significant interaction we observed may offer a novel perspective on this ongoing debate: the predictive value of RDW may depend critically on the inflammatory context in which it is measured.

To explore the biological underpinnings of this interaction, we examined the correlation structure between RDW and a panel of systemic inflammatory markers. After Benjamini–Hochberg correction, RDW exhibited significant positive correlations with IL-6, CRP, SAA, and MLR (all FDR < 0.05). These associations are mechanistically coherent. Persistent immune activation in LTBI may trigger inflammasome-mediated pathways that are centrally involved in atherogenesis (Tall and Bornfeldt, 2023). Pro-inflammatory cytokines, particularly IL-6, are potent inducers of hepatic hepcidin synthesis. Hepcidin degrades ferroportin on macrophages and duodenal enterocytes, blocking the sole cellular iron export pathway and leading to combined iron sequestration and reduced intestinal absorption (Fraenkel, 2015; Li et al., 2020). The consequent functional iron deficiency impairs hemoglobinization and disrupts erythroid maturation, manifesting as elevated RDW (Cercamondi et al., 2021). In LTBI, this cascade is chronically sustained by persistent *M. tuberculosis* antigen-driven immune activation, as evidenced by elevations in CRP and ferritin documented in LTBI populations (Pavan Kumar et al., 2025; Naik et al., 2020).

A critical distinction between RDW and conventional inflammatory markers merits emphasis. CRP and IL-6 are acute-phase reactants that fluctuate within hours to days, making single time point measurements susceptible to misclassification. RDW, by contrast, reflects cumulative erythrocyte turnover integrated over the approximately 120-day erythrocyte lifespan, a property that may confer superior stability as a risk marker in the context of chronic, low-grade inflammation characteristic of LTBI. This distinction likely explains why RDW, rather than IL-6, CRP, or SAA, emerged as an independent predictor. It should be acknowledged, however, that RDW is not specific to inflammation: conditions such as iron deficiency, nutritional deficiencies, and renal dysfunction can also elevate RDW. RDW should therefore be interpreted within the full clinical context rather than as a standalone inflammatory marker.

Taken together, these results suggest that LTBI creates a chronic, low-grade inflammatory milieu that accelerates atherogenesis (Magodoro et al., 2025), while RDW provides a cumulative hematological readout of this sustained immune activation. The observed interaction implies that RDW warrants different clinical interpretation depending on LTBI status: a borderline RDW value in a non-infected individual may reflect substantially greater CAD risk in an LTBI-positive patient. Whether this concept extends to other chronic infections linked to cardiovascular risk merits further study.

### 4.3 Clinical implications of age and HbA1c

The inclusion of age and HbA1c in the final model aligns with their established roles as cardiovascular risk determinants. LTBI-positive individuals in our cohort had significantly higher HbA1c levels than their LTBI-negative counterparts, consistent with evidence linking tuberculosis infection to glucose metabolism disturbances (Huang and Murray, 2025). This may reflect bidirectional interactions between chronic inflammation and insulin signaling. From a clinical standpoint, elevated HbA1c in an LTBI-positive patient should prompt integrated management of glycemic control and CAD prevention, rather than being dismissed as an isolated metabolic finding. Alongside RDW, which captures the inflammatory dimension, HbA1c represents the metabolic pathway through which LTBI may accelerate atherogenesis.

### 4.4 Limitations of the study

Several limitations should be acknowledged. First, the cohort consisted of patients who underwent both CCTA and IGRA for clinical indications, inherently selecting for individuals at elevated pretest probability of both coronary artery disease and tuberculosis infection. The resulting CAD prevalence (76.8%) substantially exceeds that in the general LTBI population. Predicted probabilities may therefore overestimate absolute risk when applied to unselected populations, although the relative ranking of risk should remain valid. Second, the cross-sectional and retrospective nature of this study limits causal inference regarding the temporal sequence between LTBI and incident CAD. This limitation is inherent to the study design and cannot be circumvented by the diagnostic method employed. IGRA-based diagnosis cannot establish whether LTBI predated the development of coronary atherosclerosis. Nevertheless, the RDW–CAD association and its modification by LTBI status are biologically grounded and corroborated by the correlation analyses linking RDW to IL-6, CRP, and SAA; thus, these associations are unlikely to be artifacts of residual confounding alone. Third, the external validation set contained only 71 LTBI-positive individuals, limiting subgroup-level precision. Fourth, the model identifies prevalent coronary atherosclerosis based on CCTA findings rather than predicting future cardiovascular outcomes such as myocardial infarction or mortality. CCTA in this study was used to determine the presence or absence of coronary atherosclerotic plaques; stenosis severity and functional significance were not assessed. Prospective follow-up of this cohort to develop a MACE prediction model represents an important future direction. Fifth, the single-region, two-center design limits generalizability. Finally, residual confounding by unmeasured factors cannot be excluded.

### 4.5 Conclusion

The present study provides a population-specific CAD risk prediction tool for LTBI-positive individuals, together with novel evidence for an RDW × LTBI multiplicative interaction. This interaction indicates that the prognostic value of RDW for CAD is substantially amplified in the presence of latent tuberculosis infection, a finding that may partly account for the inconsistent performance of RDW in prior cardiovascular risk studies. Whether RDW elevation temporally precedes incident CAD in LTBI individuals, and whether RDW can serve as a dynamic marker for monitoring cardiovascular risk during LTBI follow-up, remain open questions for prospective investigation. Whether LTBI treatment, targeted anti-inflammatory strategies, or interventions aimed at reducing RDW can lower subsequent CAD risk also remains to be evaluated in future interventional studies.

## Data Availability

The original contributions presented in the study are included in the article/**Supplementary Material**. Further inquiries can be directed to the corresponding author.

## References

[B1] Alsayed Hasanain A. F. El-Maghraby K. M. Zayed A. A. Nafee A. M. Abdel-Aal S. M. Bakkar S. M. (2018). Latent tuberculosis infection among patients with coronary artery stenosis: A case-control study. Int. J. Mycobacteriol 7, 143–147. doi: 10.4103/ijmy.ijmy_34_18 29900890

[B2] Cercamondi C. I. Stoffel N. U. Moretti D. Zoller T. Swinkels D. W. Zeder C. . (2021). Iron homeostasis during anemia of inflammation: A prospective study of patients with tuberculosis. Blood 138, 1293–1303. doi: 10.1182/blood.2020010562 33876222

[B3] Cui X. Gao L. Cao B. (2020). Management of latent tuberculosis infection in China: Exploring solutions suitable for high-burden countries. Int. J. Infect. Dis. 92S, S37–S40. doi: 10.1016/j.ijid.2020.02.034 32114201

[B4] Danese E. Lippi G. Montagnana M. (2015). Red blood cell distribution width and cardiovascular diseases. J. Thorac. Dis. 7, E402–E411. doi: 10.3978/j.issn.2072-1439.2015.10.04 26623117 PMC4635283

[B5] Djaharuddin I. Amir M. Qanitha A. (2023). Exploring the link between cardiovascular risk factors and manifestations in latent tuberculosis infection: A comprehensive literature review. Egypt Heart J. 75, 43. doi: 10.1186/s43044-023-00370-5 37249745 PMC10229520

[B6] Fraenkel P. G. (2015). Understanding anemia of chronic disease. Hematol. Am. Soc Hematol. Educ. Program 2015, 14–18. doi: 10.1182/asheducation-2015.1.14 26637695

[B7] Huaman M. A. De Cecco C. N. Bittencourt M. S. Ticona E. Kityo C. Ballena I. . (2021). Latent tuberculosis infection and subclinical coronary atherosclerosis in Peru and Uganda. Clin. Infect. Dis. 73, e3384-e3390. doi: 10.1093/cid/ciaa1934 33388766 PMC8563179

[B8] Huaman M. A. Ticona E. Miranda G. Kryscio R. J. Mugruza R. Aranda E. . (2018). The relationship between latent tuberculosis infection and acute myocardial infarction. Clin. Infect. Dis. 66, 886–892. doi: 10.1093/cid/cix910 29069328 PMC5850031

[B9] Huang C. C. Murray M. B. (2025). Tuberculous hyperglycaemia: A clinical, epidemiological and public health perspective. BMJ Glob. Health 10, e019261. doi: 10.1136/bmjgh-2025-019261 41360477 PMC12684084

[B10] Khoufi E. A. A. (2021). Association between latent tuberculosis and ischemic heart disease: A hospital-based cross-sectional study from Saudi Arabia. Pan Afr Med. J. 38, 362. doi: 10.11604/pamj.2021.38.362.28110 34367441 PMC8308999

[B11] Kimura H. Tanaka K. Saito H. Iwasaki T. Kazama S. Shimabukuro M. . (2023). Impact of red blood cell distribution width-albumin ratio on prognosis of patients with CKD. Sci. Rep. 13, 15774. doi: 10.1038/s41598-023-42986-2 37737253 PMC10516924

[B12] Kubalová K. Porvazník I. Majherová M. Demková L. Piotrowska A. Blaščáková M. M. (2025). Lipid levels and atherogenic indices as important predictive parameters in the assessment of cardiovascular risk in patients with pulmonary tuberculosis—Slovak pilot study. Medicina 61, 365. doi: 10.3390/medicina61030365 40142177 PMC11943598

[B13] Li Y. Huang X. Wang J. Huang R. Wan D. (2020). Regulation of iron homeostasis and related diseases. Mediators Inflamm. 2020, 6062094. doi: 10.1155/2020/6062094 32454791 PMC7212278

[B14] Li S. Zhang W. Liang X. (2023). Red blood cell distribution width and mortality risk in critically ill cardiovascular patients. Heliyon 9, e22225. doi: 10.1016/j.heliyon.2023.e22225 38045131 PMC10692801

[B15] Libby P. (2021). The changing landscape of atherosclerosis. Nature 592, 524–533. doi: 10.1038/s41586-021-03392-8 33883728

[B16] Magodoro I. M. Ntusi N. A. B. Jao J. Zar H. J. Claggett B. L. Siedner M. J. . (2025). Cardiometabolic biomarkers and systemic inflammation in US adolescents and young adults with latent tuberculosis infection: A population-based cohort study. Open Forum Infect. Dis. 12, ofaf194. doi: 10.1093/ofid/ofaf194 40242065 PMC12002011

[B17] Naik S. Alexander M. Kumar P. Kulkarni V. Deshpande P. Yadana S. . (2020). Systemic inflammation in pregnant women with latent tuberculosis infection. Front. Immunol. 11, 587617. doi: 10.3389/fimmu.2020.587617 33584652 PMC7873478

[B18] Noordermeer D. J. van Rooij F. Kavousi M. van den Bosch A. E. (2026). Red blood cell distribution width for prediction of new-onset heart failure in the general population. Int. J. Cardiol. 458, 134559. doi: 10.1016/j.ijcard.2026.134559 42134667

[B19] Pavan Kumar N. M R. Nancy A. Kumar B. S. M J. Ahamed S. F. . (2025). Acute-phase proteins as biomarkers of inflammation in HIV patients with latent tuberculosis: A prospective study. Front. Immunol. 16, 1551775. doi: 10.3389/fimmu.2025.1551775 40370442 PMC12075211

[B20] Perlstein T. S. Weuve J. Pfeffer M. A. Beckman J. A. (2009). Red blood cell distribution width and mortality risk in a community-based prospective cohort. Arch. Intern. Med. 169, 588–594. doi: 10.1001/archinternmed.2009.55 19307522 PMC3387573

[B21] Shah N. Pahuja M. Pant S. Handa A. Agarwal V. Patel N. . (2017). Red cell distribution width and risk of cardiovascular mortality: Insights from National Health and Nutrition Examination Survey (NHANES)-III. Int. J. Cardiol. 232, 105–110. doi: 10.1016/j.ijcard.2017.01.045 28117138

[B22] Su C. Liao L. Z. Song Y. Xu Z. W. Mei W. Y. (2014). The role of red blood cell distribution width in mortality and cardiovascular risk among patients with coronary artery diseases: A systematic review and meta-analysis. J. Thorac. Dis. 6, 1429–1440. doi: 10.3978/j.issn.2072-1439.2014.09.10 25364520 PMC4215144

[B23] Sumbal A. Sheikh S. M. Ikram A. Amir A. Sumbal R. Saeed A. R. (2023). Latent tuberculosis infection (LTBI) as a predictor of coronary artery disease: A systematic review and meta-analysis. Heliyon 9, e15365. doi: 10.1016/j.heliyon.2023.e15365 37089330 PMC10119758

[B24] Tall A. R. Bornfeldt K. E. (2023). Inflammasomes and atherosclerosis: A mixed picture. Circ. Res. 132, 1505–1520. doi: 10.1161/CIRCRESAHA.123.321637 37228237 PMC10213995

[B25] Tonelli M. Sacks F. Arnold M. Moye L. Davis B. Pfeffer M. . (2008). Relation between red blood cell distribution width and cardiovascular event rate in people with coronary disease. Circulation 117, 163–168. doi: 10.1161/CIRCULATIONAHA.107.727545 18172029

[B26] World Health Organization (2024). Global Tuberculosis Report 2024 (Geneva: World Health Organization). Available online at: https://www.who.int/teams/global-tuberculosis-programme/tb-reports/global-tuberculosis-report-2024 (Accessed December 1, 2025).

[B27] Zavragiu A. C. Sutoi D. Radbea O. R. Chiu B. Mailat D. E. Ardelean S. . (2025). Insights into the use of erythrocyte and platelet distribution indices for assessing the extent of coronary lesions. Medicina 61, 1939. doi: 10.3390/medicina61111939 41303776 PMC12654548

