# Supplementary material for: Red blood cell distribution width is associated with coronary artery disease in latent tuberculosis infection: a machine learning-based study
Source: Front Cell Infect Microbiol. 2026 Aug 14;16:1836144. doi: 10.3389/fcimb.2026.1836144 (PMC13522151; doi:10.3389/fcimb.2026.1836144)
Supplement: Supplementary file 1 [file DataSheet1.docx]

**Supplementary Materials**

**Distribution of comorbidity in Cohort A :**

The most prevalent comorbidities were coronary heart disease (CHD), hypertension (HP), diabetes mellitus (DM), arteriosclerosis, and chronic respiratory diseases (CRDs: COPD, asthma, pulmonary fibrosis, bronchiectasis). Among the 453 participants, 344 (76.0%) had at least one comorbidity. Specifically, 2 cases (0.4%) presented five comorbidities, 17 cases (3.8%) had four or more comorbidities, and 53 cases (11.7%) exhibited three or more comorbidities. Detailed breakdowns by LTBI and CAD status are shown in the table 1-2. The bar chart (e-Figure1) displays the frequency and proportion of each comorbidity in the derivation cohort. Hypertension was the most prevalent condition, followed by chronic respiratory diseases, and diabetes mellitus.

e-Figure 1. Distribution of Comorbidities by Group

Note: Prevalence of hypertension and diabetes mellitus was higher in both the LTBI and non-LTBI groups compared with the general population.* *P*＜0.05；** *P*＜0.01；****P*＜0.001. Abbreviations: CAD, coronary artery disease; HP, hypertension; DM, diabetes mellitus; CRD, chronic respiratory diseases; AF, atrial fibrillation;HF, heart failure; RF, respiratory failure.

e-Table 1. Univariable analysis odds ratios (ORs) for risk factors of concurrent CAD across different cohorts

| factors | Cohort A | | | LTBI | | | non-LTBI | | |
| --- | --- | --- | --- | --- | --- | --- | --- | --- | --- |
|  | OR | 95%CI | P value | OR | 95%CI | P value | OR | 95%CI | P value |
| age | 1.092 | 1.068-1.117 | ＜0.001 | 1.087 | 1.051-1.124 | ＜0.001 | 1.097 | 1.063-1.132 | ＜0.001 |
| Male | 2.439 | 1.157-3.796 | ＜0.001 | 2.159 | 1.163-4.005 | 0.015 | 2.844 | 1.505-5.374 | 0.001 |
| HP | 2.932 | 1.853-4.638 | ＜0.001 | 3.324 | 1.732-6.380 | ＜0.001 | 2.583 | 1.352-4.936 | 0.004 |
| DM | 3.400 | 1.746-6.621 | ＜0.001 | 2.817 | 1.198-6.625 | 0.018 | 4.39 | 1.495-12.888 | 0.007 |
| L | 0.847 | 0.678-1.059 | 0.145 | 0.927 | 0.728-1.180 | 0.538 | 0.659 | 0.440-0.986 | 0.042 |
| RDW | 1.481 | 1.190-1.845 | ＜0.001 | 2.099 | 1.404-3.137 | ＜0.001 | 1.232 | 0.968-1.569 | 0.090 |
| HDL | 0.219 | 0.100-0.478 | ＜0.001 | 0.222 | 0.064-0.770 | 0.018 | 0.216 | 0.078-0.597 | 0.003 |
| HbA1c | 1.484 | 1.199-1.837 | ＜0.001 | 1.495 | 1.116-2.002 | 0.007 | 1.472 | 1.075-2.016 | 0.016 |
| CRP | 1.026 | 1.009-1.046 | 0.006 | 1.039 | 1.012-1.076 | 0.012 | 1.015 | 0.993-1.041 | 0.219 |
| IL-6 | 1.007 | 0.996-1.019 | 0.229 | 1.013 | 0.990-1.037 | 0.272 | 1.005 | 0.994-1.017 | 0.372 |
| SAA | 1.005 | 1.001-1.010 | 0.017 | 1.010 | 1.001-1.019 | 0.037 | 1.003 | 0.998-1.008 | 0.211 |

e-Table 2 Multivariable Logistic Regression

|  | Coef | S.E. | Wald | OR | 95%CI | P-value |
| --- | --- | --- | --- | --- | --- | --- |
| Intercept | -10.680 | 2.909 | 13.480 |  |  |  |
| Age | 0.0764 | 0.0181 | 17.790 | 1.079 | 1.043- 1.120 | <0.001 |
| HbA1c (%) | 0.3541 | 0.1477 | 5.749 | 1.425 | 1.097- 1.964 | 0.017 |
| RDW (%) | 0.6175 | 0.2120 | 8.486 | 1.854 | 1.269 - 2.900 | 0.004 |

Coef = coefficient; S.E. = standard error; OR = odds ratio; 95% CI = 95% confidence interval.

e-Table 3 LTBI Interactions With CAD Risk Factors

| **Characteristic** | **OR** | **95% CI** | **p-value** |
| --- | --- | --- | --- |
| **LTBI** | 0.001 | 0.000,0.620 | 0.042 |
| **age** | 1.091 | 1.058,1.129 | **<0.001** |
| **RDW** | 1.067 | 0.844,1.390 | 0.604 |
| **HbA1c** | 1.242 | 0.944,1.711 | 0.149 |
| **LTBI * age** | 0.989 | 0.943,1.038 | 0.657 |
| **LTBI * RDW** | 1.737 | 1.092,2.877 | **0.025** |
| **LTBI * HbA1c** | 1.147 | 0.758,1.748 | 0.514 |

OR = Odds Ratio; CI = Confidence Interval

Bold indicates *P* < 0.05.

e-Table 4 Correlation of RDW with Other Risk Factors Across Cohorts

| factors | Full cohort | | LTBI group | | Non-LTBI group | |
| --- | --- | --- | --- | --- | --- | --- |
|  | Spearman's *r_s_* | FDR | Spearman's *r_s_* | FDR | Spearman's *r_s_* | FDR |
| AF | 0.199^***^ | ＜0.001 | 0.207 | 0.0041 | 0.196 | 0.0106 |
| age | 0.265^***^ | ＜0.001 | 0.223 | 0.0024 | 0.305^***^ | ＜0.001 |
| BMI | -0.032 | 0.5953 | 0.134 | 0.0668 | -0.197 | 0.0106 |
| COPD | -0.037 | 0.5543 | -0.04 | 0.5673 | -0.035^*^ | 0.7681 |
| CRP | 0.194^***^ | ＜0.001 | 0.251 | 0.001 | 0.135 | 0.0988 |
| DM | 0.053 | 0.3818 | 0.066 | 0.3941 | 0.045^*^ | 0.6893 |
| EOS | -0.084 | 0.1153 | -0.06 | 0.422 | -0.109 | 0.2107 |
| Hb | 0.017 | 0.7652 | 0.083 | 0.2725 | -0.063 | 0.5662 |
| HD | 0.122^*^ | 0.0201 | 0.168 | 0.0205 | 0.066 | 0.5662 |
| HP | 0.094 | 0.0791 | 0.171 | 0.0205 | 0.016^*^ | 0.9065 |
| IL6 | 0.337^***^ | ＜0.001 | 0.345^***^ | ＜0.001 | 0.329^***^ | ＜0.001 |
| L | -0.233^***^ | ＜0.001 | -0.168 | 0.0205 | -0.305^***^ | ＜0.001 |
| MHR | 0.153^*^ | 0.0026 | 0.109 | 0.1488 | 0.204 | 0.0099 |
| MLR | 0.296^***^ | ＜0.001 | 0.214 | 0.0034 | 0.378^***^ | ＜0.001 |
| N | 0.097 | 0.0745 | 0.142 | 0.054 | 0.05 | 0.6787 |
| PDW | 0.02 | 0.746 | 0.045^*^ | 0.5446 | -0.008 | 0.9088 |
| PLT | -0.001 | 0.9826 | -0.002^**^ | 0.971 | -0.01^*^ | 0.9088 |
| SAA | 0.215^***^ | ＜0.001 | 0.238 | 0.0013 | 0.191 | 0.0114 |
| WBC | 0.039 | 0.5543 | 0.104 | 0.1625 | -0.027^*^ | 0.8172 |

* *P*＜0.05；** *P*＜0.01；****P*＜0.001
